# A Novel Sampling Model to Study the Epidemiology of Canine Leishmaniasis in an Urban Environment

**DOI:** 10.3389/fvets.2021.642287

**Published:** 2021-03-08

**Authors:** Lucy A. Parker, Lucrecia Acosta, Mariana Noel Gutierrez, Israel Cruz, Javier Nieto, Enrique Jorge Deschutter, Fernando Jorge Bornay-Llinares

**Affiliations:** ^1^Departamento de Salud Pública y Ginecología, Universidad Miguel Hernández de Elche, Alicante, Spain; ^2^CIBER Epidemiología y Salud Pública, Madrid, Spain; ^3^Área de Parasitología, Departamento de Agroquímica y Medio Ambiente, Universidad Miguel Hernández de Elche, Alicante, Spain; ^4^Veterinary Centre “Dame la Pata”, Posadas, Argentina; ^5^National School of Public Health, Instituto de Salud Carlos III, Madrid, Spain; ^6^WHO Collaborating Centre for Leishmaniasis, Servicio de Parasitología, Centro Nacional de Microbiología, Instituto de Salud Carlos III, Madrid, Spain; ^7^Facultad de Ciencias Exactas, Químicas y Naturales, Universidad Nacional de Misiones, Posadas, Argentina

**Keywords:** visceral leishmaniasis, canine leishmaniasis, sampling, epidemiology, canine population

## Abstract

**Background:** Visceral leishmaniasis (VL) is one of the most important parasitic diseases in the world. The domestic dog is the main reservoir of zoonotic VL and a high prevalence of canine leishmaniasis (CanL) is associated with transmission of infection to humans. Here we describe the methodology used to obtain a rapid and representative sample of domestic dogs in the city of Posadas, Misiones, and compare the prevalence of *Leishmania* infection with a sample of shelter dogs.

**Methodology:** We used the city land registry to make a random selection of homes and systematically recruited 349 domestic dogs from the selected properties. We also included all dogs from the main canine shelter within the city. Dogs were examined by two experienced veterinarians who recorded the presence of clinical signs common in CanL using a standardized protocol. We extracted a blood sample from each dog and performed four different serological tests to reveal the presence of anti-*Leishmania* antibodies.

**Results:** After clinical examination, 145 domestic dogs (41.5%) and 63 (90%) shelter dogs had clinical signs compatible with CanL (*p* < 0.001). The seroprevalence among domestic dogs was 20.1% (95% CI 16.1–24.6) which was significantly lower than among the abandoned dogs (38.6%, 95% CI 27.7–50.6, *p* < 0.001). The spatial distribution of infected dogs was fairly homogenous throughout the city. Among domestic dogs, we observed a positive association between where the dog slept and presence of anti-*Leishmania* antibodies (*p* = 0.034). Of the seropositive domestic dogs 38 (54.4%) were asymptomatic.

**Conclusions:** Our findings demonstrate how seroprevalence results can be highly influenced by sampling methodology. We demonstrate how the land registry can be used to estimate the prevalence of CanL in representative sample of domestic dogs in an urban setting, allowing decision makers to deepen their understanding the epidemiology of CanL in a timely and efficient manner for the development of plans to address both human and canine disease.

## Introduction

Visceral leishmaniasis (VL) is one of the most important parasitic diseases in the world. The domestic dog is the main reservoir of zoonotic VL (ZVL) and a high prevalence of canine leishmaniasis (CanL) is associated with transmission of infection to humans (1, 2). ZVL is widespread in Latin America (3), where it is caused by *Leishmania infantum* (syn. *L. chagasi*) transmitted by phlebotomine sand flies of the genus *Lutzomyia* (Psychodidae) (4), and is an increasing public health problem (5).

It has a strong complex link with poverty mainly in rural and suburban areas but has been growing among urban populations in recent years (6). Emerging focuses of disease can be difficult to manage and have been associated with widespread culling of infected dogs although evidence supporting this approach is lacking (7). Many factors are thought to contribute to the expansion and urbanization of ZVL, such as environmental changes, changes in the ecology and biology of *Lutzomyia longipalpis* and population migration from rural to urban areas (8).

In Argentina, leishmaniasis is an emerging disease, with a growing number of human and canine clinical cases (9). In Posadas, in the province of Misiones, Argentina, the first human transmission of ZVL associated with dogs and *Lu. longipalpis* was reported in 2006 (10). The presence of *L. infantum* was further described in *Lu. longipalpis* sandflies and dogs using molecular methods (11, 12). In 2009, after human cases of Leishmania had been identified within the city, local health authorities were keen to understand the spread of canine infection within the city in a non-biased manner. Together with local health authorities, we set out to estimate the prevalence of *Leishmania* infection among the canine population in Posadas, by designing a rapid but robust sampling method to understand the epidemiology of the infection within the city using the available but limited resources. This current report shares the methods we used and demonstrate the importance of the adequate selection of the sample and its impact on the epidemiological interpretation of the disease.

In order to provide a valid estimation of disease prevalence it is necessary to include a representative sample of the total population. Ideally, estimates should be drawn through a simple random sample where all members of the population have an equal opportunity to be selected in the sample, but when it comes to dog populations it can be logistically difficult to determine the total population size let alone the probability of the inclusion of each animal in the sample (13). When recent canine censuses are available, prevalence studies are able to obtain a representative sample fairly easily by extracting a random sample from the census or performing the serosurvey alongside the census. Surveys of this type may be carried out due to the known presence of human or canine cases of VL (14). Even when canine censuses are present, limitations remain because they are rarely up to date and do not include free roaming dogs. Other options include the recruitment of animals through the local veterinary practices (15), or taking advantage of local rabies vaccination campaigns (16).

In Posadas, like many of the places in Latin America where CanL is widespread, there was no official census of canines that could be used to extract a random sample. Furthermore, there was a significant population of free-roaming dogs with no owner, and registration of the domestic dogs with local veterinary practices is far from comprehensive. For this reason, we needed to develop a novel strategy to extract a random sample of domestic dogs within the city, working with the information available. Furthermore, Misiones is one of the poorest provinces in Argentina and given the competing health problems in this area, we wanted to make the estimation as efficiently as possible, i.e., using the minimum number of dogs to make an accurate estimation with adequate precision.

In this paper, we describe the methodology used to obtain a representative sample of domestic dogs in the city of Posadas. We describe the presence of anti-*Leishmania* antibodies in domestic dogs and a systematic sample of dogs housed in a private dog shelter in the same region in the same time period. We identify variables related to infection in both populations. The main objective of this report is to share the methodology used to obtain a rapid and representative sample of domestic dogs in this urban setting. Furthermore, we aim to reveal how the sampling strategy can have major implications on the validity of epidemiological indicators for the development and monitoring of activities to address canine disease.

## Methods

### Study Area

The surveys were conducted between 1st of October and 15th of November 2009 in the city of Posadas (27° 23′ S, 55° 53′ W), located in the southwest of Misiones province, northeast Argentina. In 2008, Posadas had an estimated population of 297,499 inhabitants. The surface area of the city is 324 Km^2^, and it is characterized by a subtropical humid climate with an annual rainfall of 1,700 mm and an average temperature of 21.5°C.

### Sample Size

To estimate the prevalence of *Leishmania* infection in domestic dogs in Posadas, we attempted to obtain a random sample of all domestic dogs in the city. We calculated a priori that we would need between 322 and 368 dogs to provide a reliable estimate with an error margin of ± 5%, at a 95% confidence level, using Epidat 3.1 software [Jan 2006; Servizo de Epidemioloxía da Dirección Xeral de Innovación e Xestión da Saúde Pública a Consellería de Sanidade (Xunta de Galicia) and the Pan American Health Organization (PAHO), http://dxsp.sergas.es]. This calculation required the following assumptions: (i) an estimated total dog population of 100,000 and (ii) an estimated prevalence of 30–40%. These assumptions were made after consultation with local government as to the expected size of the domestic dog population and observation for the prevalence obtained in similar settings in the region (3, 12).

### Sampling Strategy

**Figure 1:**
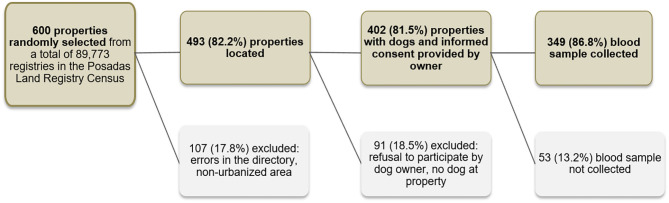
Sampling strategy used to obtain a representative sample of domestic dogs in Posadas, Argentina, 2009.

Given that it is unfeasible to carry out simple random sampling of the Posadas city domestic dogs, we used the City Land Registry Census to define our primary sampling unit. In this census, the city of Posadas is divided into almost 90,000 separate registries which mostly refer to individual properties. To allow for registry errors, non-urbanized entries on the land registry (e.g., parks, wasteland, recently flooded areas, etc.), dog owner's refusal to participate in the survey and ineligibility of the property (e.g., commercial properties or properties without a dog) we selected a total of 600 properties from the City Land Registry by simple random sampling. To minimize losses, it was decided a priori that if the initially selected property was ineligible (due to being commercial or not having a dog) the field workers would go to the residence located directly to the right. Furthermore, when more than one dog lived in the property all dogs were offered diagnosis but only one was selected at random to be included in the prevalence survey. Finally, 349 dogs were included in the survey. A detailed description of the selection process can be found in Figure 1. The location of the sampled dogs was plotted on the map of the city using a GPS point taken in the residence.

Finally, we also recruited 70 abandoned dogs from the “El Refugio” dog shelter, located in the outskirts of Posadas city. The sample constituted of all dogs present at the shelter when the study team visited. Repellent was not used at the dog shelter.

### Clinical Observation and Data Collection

Each dog was examined by two experienced veterinarians who recorded clinical signs using a standardized protocol. Dogs were considered symptomatic if they had one or more of the following signs, common in canine leishmaniasis: alopecia, desquamations, skin ulcers, alterations in oral and nasal mucosa, apathy, weight loss, cachexia, bleeding, onychogryphosis, ocular signs (conjunctivitis, uveitis or any other), lymphadenopathy, hepatomegaly and splenomegaly. Other parameters recorded were age, sex, breed, the number of dogs in the residence, and whether the dog slept inside or outside the house. Dogs were grouped according to their age in four different groups: younger than 1 year, from 1 to 4 years, from 5 to 9 years and 10 years or over.

### Biological Samples

After clinical examination 0.6 mL of peripheral blood was collected in a Multivette^®^ 600 EDTA tube (Sarstedt AG & Co., Nümbrecht, Germany). Plasma was separated by centrifugation (5 min to 3,000 r.p.m. in a bench top micro centrifuge). Blood samples were analyzed at extraction time and the rest of the samples were stored at 4°C (Universidad Nacional de Misiones, UNaM) until shipment to the WHO Collaborating Centre for Leishmaniasis (WHO-CCL) in Madrid (Spain), where they were stored at −20°C until later analysis. All analysis were performed within 4 months of sample collection.

### Detection of Anti-*Leishmania* Antibodies

Four different serological tests were performed to reveal the presence of anti-*Leishmania* antibodies, two of them based on the recombinant antigen rK39 (Kalazar Detect^®^ immunochromatographic test, rK39-ICT, and an *in-house* enzyme linked immunosorbent assay, rK39-ELISA), and the other two based on whole antigen (a commercial direct agglutination test, DAT, and *in-house* immunofluorescence antibody test, IFAT) (17). A more detailed description of these methods can be found in the Supplementary Data 1. A dog was considered seropositive when it yielded a positive result for at least one serological method using total antigen (IFAT and/or DAT) as well as one using recombinant antigen (ICT whole blood and/or plasma and/or ELISA).

### Statistical Analysis

Data recorded from the surveys were entered into an Excel (Microsoft, Redmond, WA, USA) spread sheet and imported into Stata SE version 15.0 (StataCorp LLC, USA). The proportion of seropositive dogs was calculated with a 95% confidence interval. The relationship between the characteristics of the dog and seroprevalence was evaluated using Pearson's chi-square test and differences were considered statistically significant when the *p*-value was <0.05.

## Results

Of 600 randomly selected land registries, 107 (17.8%) were excluded because of errors in the registry or because they were non-urbanized plots of land. Of 493 registries with built properties, we further excluded 91 (18.5%) because there was no dog residing at the property, or because the dog owner refused to participate in the study. Finally, of 402 dog owners who agreed to participate and signed the informed consent, 52 (13.2%) were not included in the sero-survey because they did not attend the appointment with the veterinarians where biological samples were extracted and clinical signs recorded.

Finally, 349 domestic dogs were included aged between 4 months and 16 years, with a mean age of 5 years. The 70 dogs from the shelter were older, aged between 3 months and 10 years, with a mean age of 7.3 years (*p* < 0.001). Table 1 shows the characteristics of the dogs from both survey populations. There were no significant differences in sex between the two populations, but the domestic dogs were more likely to be pure breed. After clinical examination, 145 domestic dogs (41.5%) and 63 (90%) shelter dogs had clinical signs suggestive of CanL (*p* < 0.001). The most common clinical sign observed in the dogs was lymphadenopathy, present in 124 (35.5%) of domestic dogs, and 52 (74.3%) of the shelter dogs. Other common signs observed in the dogs were onychogryphosis (51, 14.6% of domestic dogs and 43, 61.4% shelter dogs), desquamations (13.7 and 30%, respectively) and alopecia (12.6 and 52.9%, respectively).

**Table 1:** Characteristics of the dogs included in the sero-surveys according to sampling strategy.

**Symptoms**	**Representative sample of domestic dogs**	**Systematic sample of shelter dogs**	***P*-value1^a^**
	***N* (%)**	***N* (%)**	
**Sex**			0.756
Male	191 (54.9)	37 (52.8)	
Female	157 (45.1)	33 (47.1)	
**Age group**			<0.001
<1 year	21 (6.1)	2 (2.9)	
1–4 years	161 (46.5)	11 (15.7)	
5–9 years	118 (34.1)	29 (41.4)	
10+ years	46 (13.3)	28 (40.0)	
**Breed**			<0.001
Pure bred	94 (26.9)	5 (7.1)	
Mongrel	255 (73.1)	65 (92.9)	
**Clinical status**			<0.001
Asymptomatic	204 (58.4)	7 (10.0)	
Symptomatic	145 (41.5)	63 (90.0)	
*Lymphadenopathy*	124 (35.5)	52 (74.3)	<0.001
*Onychogryphosis*	51 (14.6)	43 (61.4)	<0.001
*Desquamations*	48 (13.7)	21 (30.0)	0.001
*Alopecia*	44 (12.6)	37 (52.9)	<0.001
*Weight loss*	39 (11.2)	22 (31.4)	<0.001
*Hepatomegaly*	32 (9.2)	22 (31.4)	<0.001
*Splenomegaly*	31 (8.9)	21 (30.0)	<0.001
*Ocular signs*	31 (8.9)	20 (28.6)	<0.001
*Pale oral mucosa*	28 (8.1)	14 (20.0)	0.002
*Ulcers*	25 (7.2)	13 (18.6)	0.002
*Bleeding*	11 (3.2)	4 (5.7)	0.292
*Apathy*	9 (2.6)	4 (5.7)	0.167
*Alterations in nose mucosa*	9 (2.6)	5 (7.1)	0.052
*Cachexia*	4 (1.2)	4 (5.7)	0.011
Total	349 (100)	70 (100)	

a *P-values are comparing the shelter dogs with the random sample of domestic dogs*.

**Figure 2:**
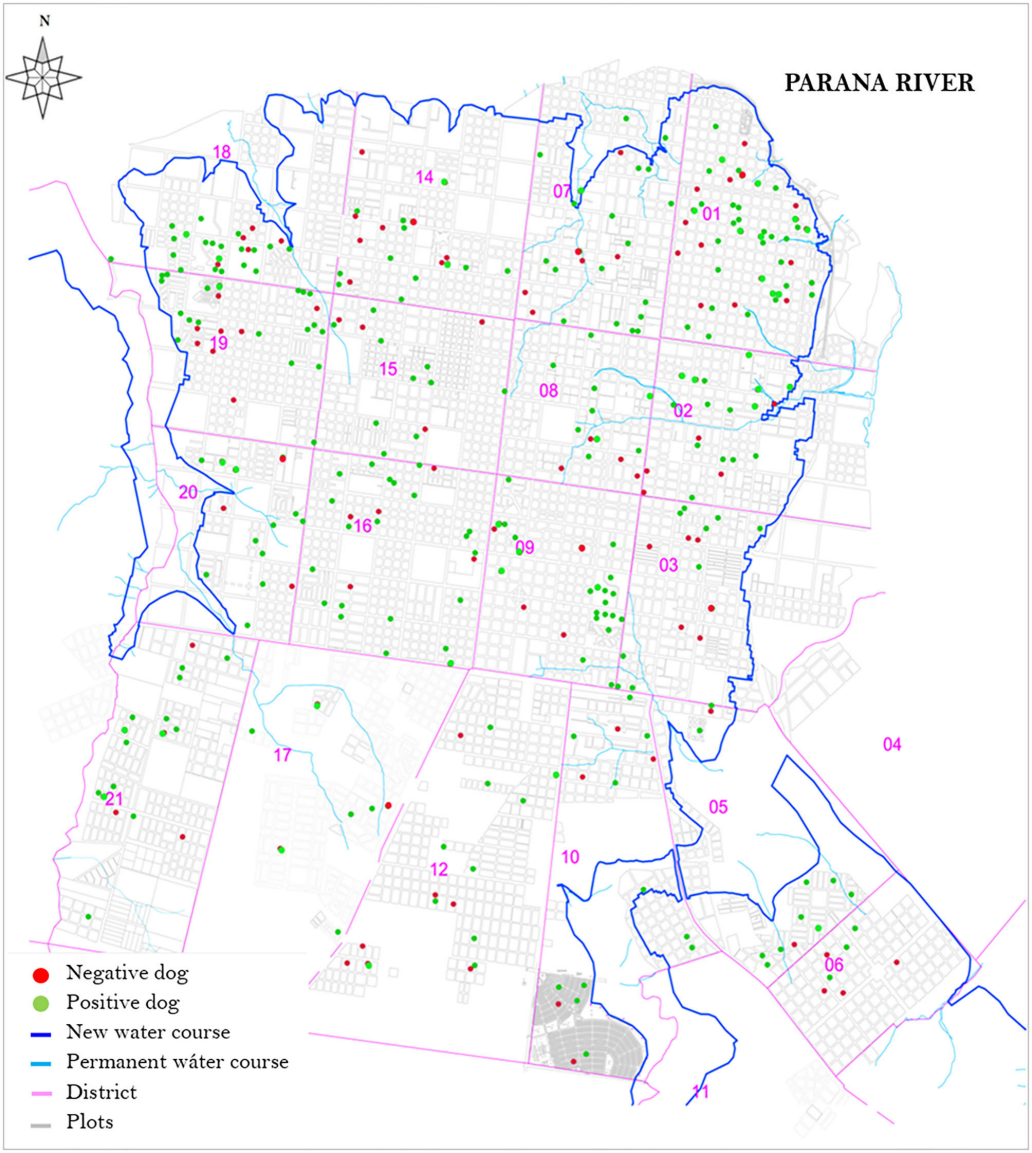
Spatial distribution of the sample of domestic dogs included in the study, according to presence of anti-*Leishmania* antibodies. New water course: New permanent water course due to construction of the Yacyretá- Apipé hydroelectric power station downstream.

The spatial distribution of the population in the study area are shown in Figure 2, along with the seroprevalence. We can observe a greater concentration of dogs, both with and without anti-*Leishmania* antibodies, in the north-east part of the city corresponding to the more densely populated city center. In less populated areas the selected dogs are more scattered.

The seroprevalence among domestic dogs was 20.1% (95% CI 16.1–24.6) which was significantly lower than among the abandoned dogs (38.6, 95% CI 27.7–50.6, *p* = 0.001). A detailed description of seroprevalence with regards to the sex, age group, breed, where dog slept, number of dogs in the house and clinical status of the dogs can be found in Table 2. We did not observe an association between seroprevalence and sex, age group and clinical status in either of the dog populations. Among domestic dogs, we observed a positive association between where the dog slept and presence of anti-*Leishmania* antibodies (*p* = 0.034). Among the dogs from the canine shelter, although there were few pure breed dogs, we observed that they were more likely to have anti-*Leishmania* antibodies (*p* = 0.048). Of the seropositive domestic dogs 38 (54.4%) were asymptomatic. Of the 204 asymptomatic domestic dogs, 38 (18.6%) were seropositive.

**Table 2:** Seroprevalence of *Leishmania* infection according to sampling strategy and characteristics of the dogs, in Posadas, Argentina 2009.

	**Representative sample of domestic dogs**			**Systematic sample of shelter dogs**		
	**Seropositivity**	**Total**	***P*-value**	**Seropositivity**	**Total**	***P*-value**
	***n* (%)**	***n***		***n* (%)**	***n* (%)**	
**Sex**			0.910			0.532
Male	38 (19.9)	191		13 (35.1)	37	
Female	32 (20.4)	157		14 (42.4)	33	
**Age group**			0.988			0.603
<1 year	4 (19.0)	21		0 (0)	2	
1–4 years	33 (20.5)	161		4 (36.4)	11	
5–9 years	23 (19.5)	118		13 (44.8)	29	
10+ years	10 (21.7)	46		10 (38.6)	28	
**Breed**			0.518			0.048
Pure bred	21 (22.3)	94		4 (80.0)	5	
Mongrel	49 (19.2)	255		23 (35.4)	65	
**Where the dog slept**			0.034			NA
Inside the house	7 (9.6)	73		–	–	
Outside the house	61 (23.2)	263		–	–	
Both	2 (15.4)	13		–	–	
**Number of dogs in residence**			0.075			NA
1	27 (15.7)	172		–	–	
2	32 (27.8)	115		–	–	
3	7 (15.9)	44		–	–	
>3	4 (22.2)	18		–	–	
**Clinical status**			0.429			0.567
Asymptomatic	38 (18.6)	204		2 (28.6)	7	
Symptomatic	32 (22.1)	145		25 (39.7)	63	
Total	70 (20.1)	349 (100)		27 (38.6)	70 (100)	

## Discussion

The prevalence of antibodies for *Leishmania* in domestic dogs from the city of Posadas was 20.1% (95% CI 16.1–24.6), suggesting that at least one in every five domestic dogs in the city is infected or has been exposed to CanL. In the sample of shelter dogs, the observed seroprevalence of CanL was nearly double at 38.6% (95% CI 27.7–50.6). Although our sampling strategy was designed to obtain a random sample of domestic dogs, and not to make geographical comparisons between areas, it was noticeable that seropositive dogs show a fairly homogeneous distribution pattern throughout the city. Because the sampling unit we used was household, we can observe a greater concentration of dogs, both with and without anti-*Leishmania* antibodies, in densely populated areas, while in less populated areas the selected dogs are more scattered. There is a stark contrast with suggestions of a relationship between the focal distribution of potential vectors in the presence of human clusters of infection in the city of Posadas (18). Even if the vector is clustered, the homogeneous distribution of the disease reservoir, perhaps associated with the high mobility of domestic dogs could have important implications for control and may play an important role in the persistence of the disease in urban settings. Other studies have shown similar high prevalence of CanL is the surrounding area which has shown a decrease in recent years (19).

While seroepidemiological surveys are commonly used to measure the prevalence of leishmaniasis, there is a lot of variation in the methods used to recruit the canine study participants, and the details of how the sampling procedures are carried out is often lacking. For example, a recent study in Colombia (20) described how the sample being carried out in municipalities with increasing numbers of clinical cases reported by health authorities, but the procedures used to access the dog population is not provided. Some studies report using veterinary practices or samples extracted during a vaccination campaign (15, 16), but it can be difficult to determine if the sample if truly representative, Confounding factors could be that dogs participating in a vaccination campaign are less likely to be infected because of the behavioral factors associated with their care. Estimating the prevalence using a canine census can derive valid estimated of domestic dog infection but canine censuses are not always available, and it can be resource heavy ensuring they are up to date. In this study we used the land registry and simple random sample to obtain a representative sample of domestic dogs.

Our study is not without limitations. On the one hand the fact that we did not attempt to include free roaming dogs should be considered a limitation as it could lead to an underestimation of the true prevalence of CanL in the city. A higher prevalence of CanL among stray dogs compared to domestic dogs has been observed in other studies (21). We can expect free roaming dogs to be more similar to shelter dogs than domestic dogs, especially if we consider the higher prevalence in dogs sleeping outside the house, which has been observed in other studies (8). We observed a markedly higher prevalence among animals recruited from the shelter. A pilot study 3 years earlier with a convenience sample had observed a prevalence of 43.6% (12). Another limitation is the incomplete sampling of shelter dogs. We recruited all dogs from “El Refugio” animal shelter, which is one of two shelters in the city, and by far the largest shelter in the city. Although it is possible that the prevalence of CanL varies between the shelters, inclusion of this shelter was made due to interest and collaboration of the shelter owner, and the disease status of the dogs was not considered when deciding which shelter to include. The dogs from the canine shelter were significantly older, and more likely to be in poor health than the domestic dogs.

Many studies have pointed out the importance of identifying asymptomatic carriers in endemic areas (22–26). Some studies show that asymptomatic dogs may comprise several between 50 and 60% of all infected dogs in the area (27–29). What is striking in this study is that despite having randomly selected the domestic dogs from a normal population a total of 54.2% of seropositive dogs were asymptomatic and only the 7.4% on canine shelter. Otherwise, 22.1% of symptomatic domestic dogs were seropositive. Therefore, a diagnosis based only on the appearance of clinical signs related to the disease could overestimate the number of cases infected by *Leishmania* in this area.

We must also acknowledge that the study was carried out in 2009, which is a major limitation if one objective is to know the prevalence of infection in the city, but the purpose of this report here is to describe our methods and demonstrate the importance of the adequate sample and its impact on the epidemiological interpretation of the disease. We did not collect information about the use of insecticide. Some studies have shown how this can influence disease presence and it should be considered in future studied (30, 31).

We did not use a multi-stage or stratified sampling strategy because we were interested in observing the geographical spread of the disease in the entire city. We could have improved the efficiency of our strategy by undertaking a multistage sampling strategy where we take a first stage random sample of larger geographical units (sectors or parishes), and then sample a specific number of land registries within this sample. This would be logistically simpler and reduce the number of movements of the team within the city, which can be important especially when the geographical area is large. In this study we used the land registry census as our primary sampling unit, and there was a significant proportion of errors in the registry. Another attractive option would be to carry out geospatial sampling and obtain a the random selection of properties or animals using randomly generated GPS points or grid-squares (19, 32, 33). Such methods are highly valid but require users to be competent in using Geographical Information Systems (GIS) which may be a challenge in many low-resource settings. Here we propose a simpler strategy using available registries which can allow local stakeholders to obtain a valid estimation of disease prevalence with limited technology.

In response to the results from the study and the model used for epidemiological investigation of the disease, preventative and curative services for canine disease were strengthened throughout the municipality of Posadas. The local government implemented a control program where dog owners from low-income families were offered a free screening service for canine leishmaniasis. It is estimated that 40% of the dog owner population took part in the scheme. More recently, the city established a Centre for Vectorial and Zoonotic Diseases, with the support of the Municipality and the Ministry of Health. Tackling CanL in the city is a key line of work within the center.

## Conclusions

Overall, the two prevalence estimations observed and the spatial distribution of the domestic dogs with anti-*Leishmania* antibodies suggest that this focus of disease was well-established in 2009. Our novel sampling strategy allowed us to obtain a representative sample of domestic dogs in an urban setting. The prevalence of CanL obtained was much lower than the prevalence among dogs from the animal shelter. Although this difference is not surprising, it is important to acknowledge how seroprevalence results are influenced by sampling methodology, and the fact that samples using convenience sampling may under or overestimate disease prevalence and can lead to misinformed decisions about control and management. We demonstrate how a representative sample can be obtained in a timely and efficient manner, allowing decision makers to deepen their understanding the epidemiology of CanL for the development of plans to address both human and canine disease, while protecting animal welfare.

## Data Availability Statement

The raw data supporting the conclusions of this article will be made available by the authors, without undue reservation.

## Ethics Statement

The animal study was reviewed and approved by the bioethics committee of the Ministry of Health in Misiones, Argentina. (Expediente: 6106-135-08); Comité de Bioética, División de Zoonosis de la Subsecretaría de Atención Primaria y Salud Ambiental Salud del Ministerio de Salud de Misiones; Resolución Ministerial No.: 2332/2008). Written informed consent was obtained from the owners for the participation of their animals in this study.

## Author Contributions

IC, LA, and FB-L conceived the study. LA, LP, FB-L, and IC designed the study. LA and LP drafted the original draft, analyzed data and realized formal analysis and interpretation of data. IC and FB-L critically reviewed it and contributed to its design. FB-L and ED supervised and coordinated the study. LA, MG, and ED contacted dog owners to enroll the study. LA, MG, JN, FB-L, and IC carried out clinical examination and obtained biological samples from the dogs. JN and MG designed the protocol for clinical scoring of the dogs. IC, LA, and JN performed serological diagnosis. All authors revised and edited the different draft versions, and read and approved the submitted manuscript.

## Conflict of Interest

The authors declare that the research was conducted in the absence of any commercial or financial relationships that could be construed as a potential conflict of interest.

## References

[1] Fraga DBM Solcà MS Silva VMG Borja LS Nascimento EG Oliveira GGS. Temporal distribution of positive results of tests for detecting Leishmania infection in stray dogs of an endemic area of visceral leishmaniasis in the Brazilian tropics: a 13 years survey and association with human disease. Vet Parasitol. (2012) 190:591–4. 10.1016/j.vetpar.2012.06.025 22795669

[2] Moreno J Alvar J. Canine leishmaniasis: epidemiological risk and the experimental model. Trends Parasitol. (2002) 18:399–405. 10.1016/S1471-4922(02)02347-4 12377257

[3] Dantas-Torres F. Canine leishmaniosis in South America. Parasit Vectors. (2009) 2 (Suppl. 1):S1. 10.1186/1756-3305-2-S1-S1 PMC2679393 19426440

[4] Lanzaro GC Ostrovska K Herrero MV Lawyer PG Warburg A. Lutzomyia longipalpis is a species complex: genetic divergence and interspecific hybrid sterility among three populations. Am J Trop Med Hyg. (1993) 48:839–47. 10.4269/ajtmh.1993.48.839 8333579

[5] Pan American Health Organization. Manual of Procedures for Leishmaniases Surveillance and Control in the Americas. Washington, DC: Pan American Health Organization (2019). Available online at: https://iris.paho.org/handle/10665.2/51838 (accessed December 2, 2020).

[6] Shaw J. The leishmaniases–survival and expansion in a changing world. A mini-review. Mem Inst Oswaldo Cruz. (2007) 102:541–7. 10.1590/S0074-02762007000500001 17899628

[7] Dantas-Torres F Miró G Baneth G Bourdeau P Breitschwerdt E Capelli G. Canine leishmaniasis control in the context of One Health. Emerg Infect Dis. (2019) 25:1–4. 10.3201/eid2512.190164 31742505 PMC6874277

[8] Leal GG de A Carneiro M Pinheiro A da C Marques LA Ker HG Reis AB. Risk profile for Leishmania infection in dogs coming from an area of visceral leishmaniasis reemergence. Prev Vet Med. (2018) 150:1–7. 10.1016/j.prevetmed.2017.11.022 29406075

[9] Salomón OD Sosa Estani S Rossi GC Spinelli GR. Lutzomyia longipalpis and leishmaniasis visceral in Argentina. Medicina. (2001) 61:174–8. 11374140

[10] Salomon O Sinagra A Nevot M Barberian G Paulin P Estevez J. First visceral leishmaniasis focus in Argentina. Mem Inst Oswaldo Cruz. (2008) 103:109–11. 10.1590/S0074-02762008000100018 18368242

[11] Acardi SA Liotta DJ Santini MS Romagosa CM Salomón OD. Detection of *Leishmania infantum* in naturally infected *Lutzomyia longipalpis* (Diptera: Psychodidae: Phlebotominae) and Canis familiaris in Misiones, Argentina: the first report of a PCR-RFLP and sequencing-based confirmation assay. Mem Inst Oswaldo Cruz. (2010) 105:796–9. 10.1590/S0074-02762010000600011 20944995

[12] Cruz I Acosta L Gutiérrez MN Nieto J Cañavate C Deschutter J. A canine leishmaniasis pilot survey in an emerging focus of visceral leishmaniasis: Posadas (Misiones, Argentina). BMC Infect Dis. (2010) 10:342. 10.1186/1471-2334-10-342 21122107 PMC3002360

[13] Belo VS Werneck GL da Silva ES Barbosa DS Struchiner CJ. Population estimation methods for free-ranging dogs: a systematic review. PLoS ONE. (2015) 10:e0144830. 10.1371/journal.pone.0144830 26673165 PMC4684217

[14] Lopes JV Michalsky ÉM Pereira NCL Paula AJV de Souza AGM Pinheiro LC. Canine visceral leishmaniasis in area with recent Leishmania transmission: prevalence, diagnosis, and molecular identification of the infecting species. Rev Soc Bras Med Trop. (2020) 53:e20200141. 10.1590/0037-8682-0141-2020 32935783 PMC7491567

[15] Velez R Ballart C Domenech E Abras A Fernández-Arévalo A Gómez SA. Seroprevalence of canine Leishmania infantum infection in the Mediterranean region and identification of risk factors: the example of North-Eastern and Pyrenean areas of Spain. Prev Vet Med. (2019) 162:67–75. 10.1016/j.prevetmed.2018.10.015 30621900

[16] Pires H Martins M Matos AC Cardoso L Monteiro F Roque N. Geospatial analysis applied to seroepidemiological survey of canine leishmaniosis in east-central Portugal. Vet Parasitol. (2019) 274:108930. 10.1016/j.vetpar.2019.108930 31586700

[17] Bray RS. Immunodiagnosis of leishmaniasis. In: Chang KP Bray RS, editors. Leishmaniasis. Amsterdam, The Netherlands: Elsevier Ltd. (1985). p. 177–182.

[18] Fernández MS Salomón OD Cavia R Perez AA Acardi SA Guccione JD. Lutzomyia longipalpis spatial distribution and association with environmental variables in an urban focus of visceral leishmaniasis, Misiones, Argentina. Acta Trop. (2010) 114:81–7. 10.1016/j.actatropica.2010.01.008 20096256

[19] Lamattina D Berrozpe PE Casas N Moya SL Giuliani MG Costa SA. Twice upon a time: the progression of canine visceral leishmaniasis in an Argentinean city. Munderloh UG, editor. PLoS ONE. (2019) 14:e0219395. 10.1371/journal.pone.0219395 31276573 PMC6611631

[20] Picón Y Almario G Rodríguez V Garcia NV. Seroprevalence, clinical, and pathological characteristics of canine leishmaniasis in a central region of Colombia. J Vet Res. (2020) 64:85–94. 10.2478/jvetres-2020-0011 32258804 PMC7105987

[21] Shokri A Fakhar M Teshnizi SH. Canine visceral leishmaniasis in Iran: a systematic review and meta-analysis. Acta Trop. (2017) 165:76–89. 10.1016/j.actatropica.2016.08.020 27570207

[22] Alvar J Cañavate C Molina R Moreno J Nieto J. Canine leishmaniasis. Adv Parasitol. (2004) 57:1–88. 10.1016/S0065-308X(04)57001-X 15504537

[23] Dantas-Torres F Brandão-Filho SP. Visceral leishmaniasis in Brazil: revisiting paradigms of epidemiology and control. Rev Inst Med Trop Sao Paulo. (2006) 48:151–6. 10.1590/S0036-46652006000300007 16847505

[24] Chargui N Amro A Haouas N Schönian G Babba H Schmidt S. Population structure of Tunisian *Leishmania infantum* and evidence for the existence of hybrids and gene flow between genetically different populations. Int J Parasitol. (2009) 39:801–11. 10.1016/j.ijpara.2008.11.016 19211023

[25] Rondon FCM Bevilaqua CML Franke CR Barros RS Oliveira FR Alcântara AC. Cross-sectional serological study of canine Leishmania infection in Fortaleza, Ceará state, Brazil. Vet Parasitol. (2008) 155:24–31. 10.1016/j.vetpar.2008.04.014 18565676

[26] Solano-Gallego L Koutinas A Miró G Cardoso L Pennisi MG Ferrer L. Directions for the diagnosis, clinical staging, treatment and prevention of canine leishmaniosis. Vet Parasitol. (2009) 165:1–18. 10.1016/j.vetpar.2009.05.022 19559536

[27] Abranches P Silva-Pereira MC Conceição-Silva FM Santos-Gomes GM Janz JG. Canine leishmaniasis: pathological and ecological factors influencing transmission of infection. J Parasitol. (1991) 77:557–61. 10.2307/3283159 1865262

[28] Solano-Gallego L Riera C Roura X Iniesta L Gallego M Valladares JE. Leishmania infantum-specific IgG, IgG1 and IgG2 antibody responses in healthy and ill dogs from endemic areas. Evolution in the course of infection and after treatment. Vet Parasitol. (2001) 96:265–76. 10.1016/S0304-4017(00)00446-5 11267753

[29] Brandonisio O Carelli G Ceci L Consenti B Fasanella A Puccini V. Canine leishmaniasis in the Gargano promontory (Apulia, South Italy). Eur J Epidemiol. (1992) 8:273–6. 10.1007/BF00144813 1644148

[30] Gálvez R Miró G Descalzo MA Nieto J Dado D Martín O. Emerging trends in the seroprevalence of canine leishmaniosis in the Madrid region (central Spain). Vet Parasitol. (2010) 169:327–34. 10.1016/j.vetpar.2009.11.025 20031330

[31] Martín-Martín I Molina R Rohoušová I Drahota J Volf P Jiménez M. High levels of anti-Phlebotomus perniciosus saliva antibodies in different vertebrate hosts from the re-emerging leishmaniosis focus in Madrid, Spain. Vet Parasitol. (2014) 202:207–16. 10.1016/j.vetpar.2014.02.045 24629428

[32] Rinaldi L Biggeri A Carbone S Musella V Catelan D Veneziano V. Canine faecal contamination and parasitic risk in the city of Naples (southern Italy). BMC Vet Res. (2006) 2:29. 10.1186/1746-6148-2-29 16995934 PMC1590007

[33] Barnard S Ippoliti C Di Flaviano D De Ruvo A Messori S Giovannini A. Smartphone and GPS technology for free-roaming dog population surveillance - a methodological study. Vet Ital. (2015) 51:165–72. 10.12834/VetIt.233.2163.3 26455368

