# Supplementary material for: A Novel Sampling Model to Study the Epidemiology of Canine Leishmaniasis in an Urban Environment
Source: Front Vet Sci. 2021 Mar 8;8:642287. doi: 10.3389/fvets.2021.642287 (PMC7982517; doi:10.3389/fvets.2021.642287)
Supplement: Supplementary file 1 [file Data_Sheet_1.docx]

Supplementary Material

# Detailed methods of the serological tests:

Detection of anti-Leishmania antibodies in peripheral blood samples was conducted directly in the households during the survey. Whole blood samples were tested with an rK39 immunchromatographic test (rK39-ICT), Kalazar Detect™ Rapid Test for Visceral Leishmaniasis (InBios International, Inc. Seattle, WA, USA), using 20 µL and according to the instructions provided by the manufacturer.

Blood samples were transferred from the household to the University of Posadas for plasma separation. The plasma fraction was shipped to the WHO Collaborating Centre for Leishmaniasis, Spain, for further serological testing, as indicated below:

Kalazar Detect™ Rapid Test (rK39-ICT) for Visceral Leishmaniasis, using 20 µL of plasma and following the instructions provided by the manufacturer.

In house rK39- enzyme linked immunosorbent assay (rK39-ELISA), following the protocol described by Gálvez et al [2010]. Briefly, 10 µl of plasma tested with rK39 antigen coated plates. The microplates were coated with 50 ng of recombinant rK39 antigen in 50 µl of coating buffer (15 mM Na2CO3, 28 mM NaHCO3, pH= 9,6) and incubated overnight at 4 ◦C. The plates were then blocked in 200 µl of buffer (Falcon Probind #3915, PBS (pH= 7.4) with 1% Tween 20) during 1 h at room temperature and later washed tree times with 150 µl PBS and 0.1% Tween20®. After a further wash, 50 µl of serum sample diluted in PBS and 0.1% tween20® (concentration 1:100) was added and the plates were incubated for a further 30 minutes (min) at room temperature. After incubation, the plates were washed again and 50 µl of anti-IgG dog peroxidase conjugate (antibody HRP conjugated, Bethyl Laboratories, INC, TX 77356) were added and incubated at room temperature during 30 min. The washing process was repeated and 50 µl of substrate buffer was added (ABTS (2,2´-Acino-bis (3-etilbenzotiazolin-6-sulfónic ácid) diammonium salt 10 mg (Sigma) in 20 ml of citrate buffer (o-phenylene diamine phosphate-citrate buffer (Sigma®) in 100 ml of distillate water) and incubated during 30 min at room temperature. The reaction was stopped by the addition of 10% SDS (100 μl). Absorbance was measured at 405 nm using an automatic reader (Merck ELISA System MIOS, Merck®). The threshold for positivity was determined with serum from 25 dogs that tested negative for all diagnostic methods, using as a reference the mean plus 3 times the standard deviation obtained for the group (the resulting optical density was 0.13).

In house immunofluorescence antibody test (IFAT), as described by Bray [1985]. Briefly, 10 μL of fold serial dilutions 1:40 (2 μL of plasma in 80 μL 1x PBS) to 1:20,480 applied onto IFAT wells containing 10 μL of 2 x 107 Leishmania infantum promastigotes/mL in 1x PBS, L. infantum reference strain MHOM/FR/78/LEM-75. Using 10 µl of FITC-conjugated rabbit anti-dog IgG (H+L) (ICN Laboratories. Plaza, Costa Mesa, California) with fluorescein isothiocyanate in Evans blue 0,01%. Finally, the slides were read with 40X objective under fluorescence microscope (Dialux 20EB, Leitz, Germany). The threshold for positivity was established at 1/160.

Direct agglutination test (DAT), using 50 µl of Leishmania freeze-dried antigen (ITMA-DAT/VL, Prince Leopold Institute of Tropical Medicine, Antwerp, Belgium). Briefly, 50 µl plasma tested using the screening method according to the manufacturer’s protocol. Titres of ≥1:1600 were considered positive.
